# Cerebral Venous Oxygen Saturation in Hypoperfusion Regions May Become a New Imaging Indicator to Predict the Clinical Outcome of Stroke

**DOI:** 10.3390/life12091312

**Published:** 2022-08-26

**Authors:** Fengqiu Cao, Mingming Wang, Shengyu Fan, Shanhua Han, Yingwei Guo, Asim Zaman, Jia Guo, Yu Luo, Yan Kang

**Affiliations:** 1 College of Medicine and Biological Information Engineering, Northeastern University, Shenyang 110169, China; 2 College of Health Science and Environmental Engineering, Shenzhen Technology University, Shenzhen 518118, China; 3 Department of Radiology, Shanghai Fourth People’s Hospital Affiliated to Tongji University School of Medicine, Shanghai 200434, China; 4 Engineering Research Centre of Medical Imaging and Intelligent Analysis, Ministry of Education, Shenyang 110169, China; 5 Department of Psychiatry, Columbia University, New York, NY 10027, USA; 6 School of Applied Technology, Shenzhen University, Shenzhen 518060, China

**Keywords:** stroke, hypoperfusion, cerebral venous oxygen saturation, unfavorable clinical outcome, imaging predictor

## Abstract

To automatically and quantitatively evaluate the venous oxygen saturation (SvO2) in cerebral ischemic tissues and explore its value in predicting prognosis. A retrospective study was conducted on 48 AIS patients hospitalized in our hospital from 2015–2018. Based on quantitative susceptibility mapping and perfusion-weighted imaging, this paper measured the cerebral SvO2 in hypoperfusion tissues and its change after intraarterial rt-PA treatment. The cerebral SvO2 in different hypoperfusion regions between the favorable and unfavorable clinical outcome groups was analyzed using an independent *t*-test. Relationships between cerebral SvO2 and clinical scores were determined using the Pearson correlation coefficient. The receiver operating characteristic process was conducted to evaluate the accuracy of cerebral SvO2 in predicting unfavorable clinical outcomes. Cerebral SvO2 in hypoperfusion (Tmax > 4 and 6 s) was significantly different between the two groups at follow-up (*p* < 0.05). Cerebral SvO2 and its changes before and after treatment were negatively correlated with clinical scores. The positive predictive value, negative predictive value, accuracy, and area under the curve of the cerebral SvO2 were higher than those predicted by the ischemic core. Therefore, the cerebral SvO2 of hypoperfusion regions was a stronger imaging predictor of unfavorable clinical outcomes after stroke.

## 1. Introduction

Acute ischemic stroke (AIS) is one of the diseases with the highest disability and mortality rate in the world, and the age of onset is becoming younger year by year [1]. The goal of ischemic stroke treatment is to establish blood flow to realize the reperfusion of ischemic tissue to save the brain tissue without necrosis [2]. As is well known, the clinical outcome of AIS patients becomes unfavorable if the brain function viability decreases [3]. Therefore, it is essential to accurately evaluate the viability of ischemic tissue and predict the risk after treatment according to image information.

In the past two decades, the perfusion mismatch ratio of computed tomography (CT) or magnetic resonance (MR) has been widely used to evaluate ischemic tissue [4]. However, there is evidence that this method is over-valued [5]. Recently, more studies proposed that the oxygen metabolism is the key to determining tissue viability after stroke, and that cerebral SvO2 can more directly evaluate the brain tissue [6,7,8]. However, there is a lack of quantitative analysis of the cerebral SvO2 in ischemic brain tissue, which may be limited by the current measurement technology of oxygen metabolism parameters.

The quantitative measurement of cerebral SvO2 is one of the challenges, especially in patients with cerebrovascular diseases. Some methods for measuring cerebral oxygen metabolism parameters based on MRI technology have been proposed, including blood oxygenation level-dependent (BOLD) methods, T2*-weighted imaging, T2-relaxation-under-spin-tagging (TRUST), and phase difference methods [9,10,11,12] These non-invasive methods can measure SvO2 in patients with stroke or other neurological diseases as well as in healthy people. Although BOLD is widely used in neuroscience, its spatial resolution is relatively low at the 1.5 Tesla field. Additionally, other measurement techniques are also limited by the non-uniformity of the macro field, diffusion of water molecules, and the network structure of blood vessels.

Alternatively, quantitative susceptibility mapping (QSM) can provide quantitative information about cerebral oxygen metabolism [13]. QSM has the advantages of high resolution and imaging is not limited to the direction of blood flow and vascular network structure. Asymmetrically prominent cortical vein (APCV) regions (with the venous vessels increasing in number and susceptibility) are often found in the stroke hemisphere on QSM images. Its appearance is related to the decrease in local SvO2 and suggests that the prognosis of patients is poor [14,15].

In recent years, QSM was used to measure cerebral SvO2 in APCV regions of stroke patients and to study the correlation between cerebral SvO2 changes and prognosis [16,17,18]. Some studies investigated the consistency between the APCV volume on QSM and the hypoperfusion volume on the time to maximum peak (Tmax) map [19,20]. In addition, some researchers also investigated the correlation between cerebral SvO2 and perfusion status [21,22]. However, no studies have been conducted on the relationship between SvO2 in hypoperfusion regions and clinical outcomes in AIS patients based on QSM measurement.

Therefore, this study aimed to propose an automated method to quantitatively measure cerebral SvO2 in hypoperfusion regions (Tmax > 4, 6, 8, 10 s) based on QSM and perfusion-weighted imaging (PWI). The correlation between cerebral SvO2 and NIHSS (National Institutes of Health Stroke Scale) scores and 90-day modified Rankin Scale (mRS) scores were also studied. We investigated whether local cerebral SvO2 in hypoperfusion can be used as an imaging indicator to provide vital information for predictions of the clinical outcome of stroke.

## 2. Materials and Methods

### 2.1. Patients

**Figure 1:**
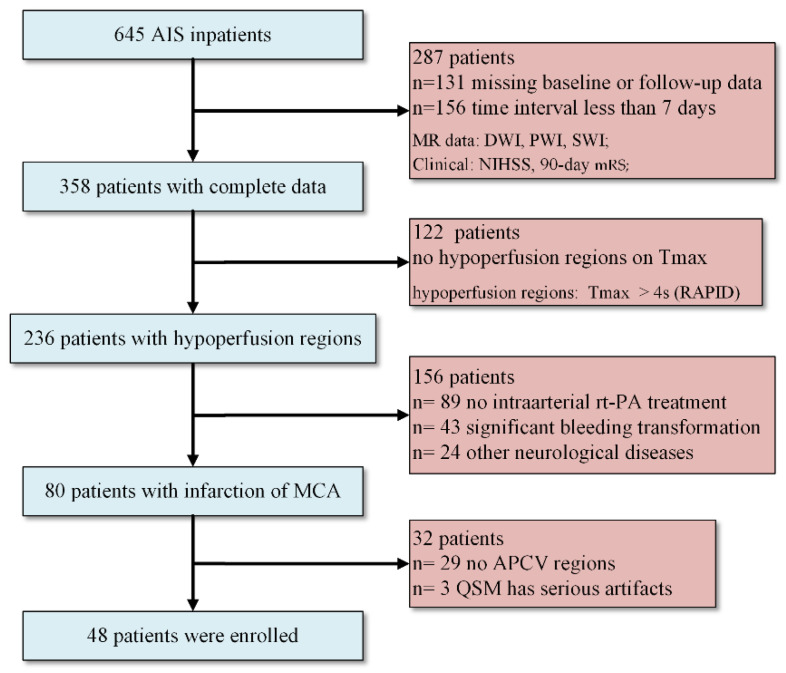
Flowchart of patient inclusion and exclusion criteria. AIS: acute ischemic stroke; DWI: diffusion-weighted imaging; PWI: perfusion-weighted imaging; SWI: susceptibility-weighted imaging; Tmax: time to maximum peak; MCA: middle cerebral artery; APCV: asymmetrically prominent cortical veins; NIHSS: National Institutes of Health Stroke Scale; mRS: modified Rankin Scale.

A retrospective study was conducted with 645 AIS patients hospitalized in the Department of Neurology of our hospital from 2015 to 2018. Patients underwent baseline MR examination within 24 h after symptom onset, and the follow-up examination was performed at discharge. Finally, 48 cases were included in the study (Figure 1). A favorable clinical outcome was defined as a 90-day mRS from 0 to 2, and an unfavorable one was defined as mRS scores from 3 to 6.

### 2.2. Imaging Protocol

All MRI data were completed on a 1.5-Tesla scanner with 20 channel coils (MAGNETOM Avanto, Siemens Healthcare, Erlangen, Germany). Table 1 summarizes the parameter settings of all sequence acquisitions. Gadopentetate dimeglumine (Shanghai Pharmaceutical Corporation, Shanghai, China) was injected with a dose of 0.2 mmol/kg body weight and a saline flush of 30 mL at the same injection flow rate of 4 mL/s.

### 2.3. QSM Reconstruction

When reconstructing QSM from phase and amplitude images, unwrapping phase information and removing background fields helped to remove the artifacts caused by the skull interface. The QSM image used the unique susceptibility inversion algorithm to solve the problem that the direction of blood flow limits the imaging, so it could more comprehensively display the distribution of cerebral veins [23]. QSM images were reconstructed by using SPIN software (v: 2.0.3; Detroit, MI, USA) in our study. A maximum intensity projection (MIP) image was generated over 16 slices of QSM data to display abnormal cortical veins.

### 2.4. Perfusion Data Processing

We used RAPID software (v2017; iSchemaView, Menlo Park, CA, USA) for the post-processing analysis of perfusion data. Hypoperfusion regions were masked on Tmax with different thresholds (4 s, 6 s, 8 s, and 10 s.) to calculate AIS patients’ local venous oxygen saturation. The volume of hypoperfusion regions (Tmax > 6 s) and the ischemic core volume (ADC < 0.62 × 10^−3^ mm^2^/s) were also measured.

**Figure 2:**
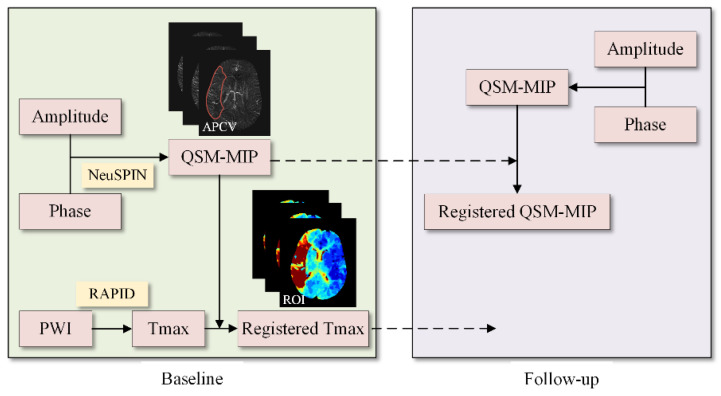
Automatic image data processing flow chart. A patient with hypoperfusion and asymmetrically prominent cortical vein (APCV) regions at baseline.

The local mutual information fast registration method was adopted to register the baseline PWI with the baseline QSM image. To compare the changes of cerebral SvO2 in hypoperfusion tissue after treatment, the follow-up QSM and the baseline QSM images needed to be registered. The hypoperfusion region on baseline Tmax images was mapped to baseline and follow-up QSM images by the transformation matrix to automatically label the region of interest (ROI) on the ischemic side (Figure 2).

### 2.5. Cerebral SvO2 Calculation

The local cerebral SvO2 can be calculated by using the susceptibility difference between cerebral veins ($X_{vein}$) and surrounding tissues ($X_{tissue}$) [24]. We assumed that the susceptibility of brain tissue without veins was equal to 0, so a threshold of 90 ppb was introduced to eliminate the susceptibility of non-blood tissue ($X_{vein-tissue}=X_{vein}$) [25]. The constants in Equation (1) were canceled out by the ratio of the change of cerebral SvO2 ($\Delta \mathrm{SvO}2_{\left(ROI\right)}=\mathrm{SvO}2_{\left(Ref\right)}-\mathrm{SvO}2_{\left(ROI\right)}$) to the cerebral SvO2 in the contralateral reference area ($\mathrm{SvO}2_{\left(Ref\right)}$) of the midline of the brain.

(1) $$X_{vein-tissue}=K\cdot {\Delta \chi }_{do}\cdot Hct\left(1-\mathrm{SvO}2\right)$$

(2) $$\Delta \mathrm{SvO}2_{\left(ROI\right)}=-\left(1-\mathrm{SvO}2_{\left(Ref\right)}\right)\cdot \left(X_{vein\left(Ref\right)}-X_{vein\left(ROI\right)}\right)∕X_{vein\left(Ref\right)}$$

where K depending on the physical properties of the magnetic field. ${\Delta \chi }_{do}$ is a constant (4π × 0.27 ppm), and it is equal to the difference in susceptibility per unit hematocrit between totally oxygenated blood and deoxygenated blood [26]. Hct means the value of hematocrit fraction in large draining veins (37–50%) [27].

For stroke patients with hypoperfusion in only one hemisphere, there was no change in cerebral SvO2 in the healthy hemisphere ($\mathrm{SvO}2_{\left(Ref\right)}$ = 70%) [28]. In order to cover most of the cortical veins and eliminate the areas prone to iron deposition in the brain, we selected all the slices containing hypoperfusion areas (ROIs) from the top slice to the middle slice of the brain to calculate the average value of cerebral SvO2.

### 2.6. Statistical Analysis

This paper used SPSS (v 26.0, International Business Machines Corporation, Armonk, America) software for all statistical analyses. Percentages were used to describe categorical variables, while mean and standard deviation were used to describe normally distributed continuous variables. Independent sample *t*-test and chi-square test were calculated to compare statistical differences of the continuous and categorical variables between favorable and unfavorable clinical outcome groups. Pearson’s analysis was performed to determine the relationship between cerebral SvO2 in different hypoperfusion regions (and its changes after treatment) and NIHSS and 90-day mRS scores. A paired *t*-test was used to verify that cerebral SvO2 can be used as an independent imaging parameter to predict the prognosis of AIS patients. Receiver operating characteristic (ROC) curves were conducted to calculate the positive predictive value, negative predictive value, diagnostic accuracy, and the area under curve (AUC) of different indicators (NIHSS, ischemic core volume, and cerebral SvO2) in discriminating unfavorable clinical outcomes. *p* < 0.05 was considered statistically significant.

## 3. Results

### 3.1. Patient Characteristics

Forty-eight patients with middle cerebral artery infarction in our hospital were recruited, including 34 male and 14 female patients. Their age ranged from 52 to 89 years, with an average age of 70.8 ± 10.2 years. The mean interval between the baseline and follow-up was 11.6 ± 4.8 days. NIHSS scores at baseline and follow-up were 7.7 ± 6.2 and 3.8 ± 4.6, respectively, and 90-day mRS scores were 1.8 ± 1.9. Among the 48 patients, there were 18 (37.5%) in the unfavorable clinical outcome group (mRS scores > 2) and 30 (62.5%) in the favorable clinical outcome group. Table 2 summarizes the baseline and follow-up characteristics of patients in different groups.

We found that the difference between the two groups was that patients in the favorable clinical outcome group at follow-up had lower NIHSS scores (1.6 ± 2.0 vs. 7.4 ± 5.2, *p* = 0.000), lower hypoperfusion volume (18.8 ± 42.0 mL vs. 69.9 ± 98.0 mL, *p* = 0.048) and greater NIHSS changes (5.4 ± 6.6 vs. 1.5 ± 5.9, *p* = 0.047). There was no significant difference in other patient characteristics between the favorable and unfavorable outcome groups.

### 3.2. Comparison of SvO2 between Two Groups

**Figure 3:**
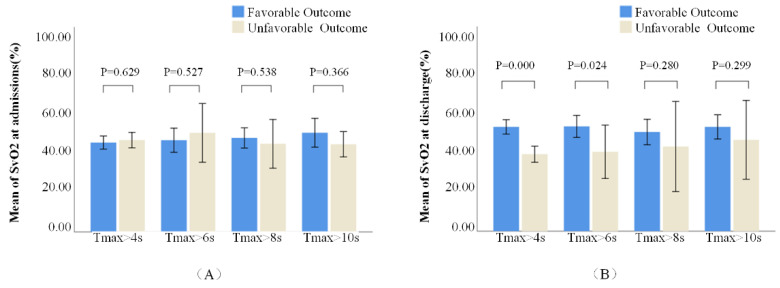
Comparison of cerebral venous oxygen saturation (SvO2) measurements in different hypoperfusion regions at baseline in panel (**A**) and follow-up in panel (**B**).

The cerebral SvO2 in different hypoperfusion regions (Tmax > 4 s, 6 s, 8 s, and 10 s) of patients with favorable and unfavorable outcomes is shown in Table 3. There was no significant difference in cerebral SvO2 between the two groups at baseline (Figure 3A). However, there were significant differences in cerebral SvO2 and its changes measured in the 4 s and 6 s regions at follow-up between the two groups (Figure 3B). We found that only 17 patients had severe hypoperfusion (Tmax > 8 s) at baseline.

### 3.3. Cerebral SvO2 Correlated with Clinical Outcomes

**Figure 4:**
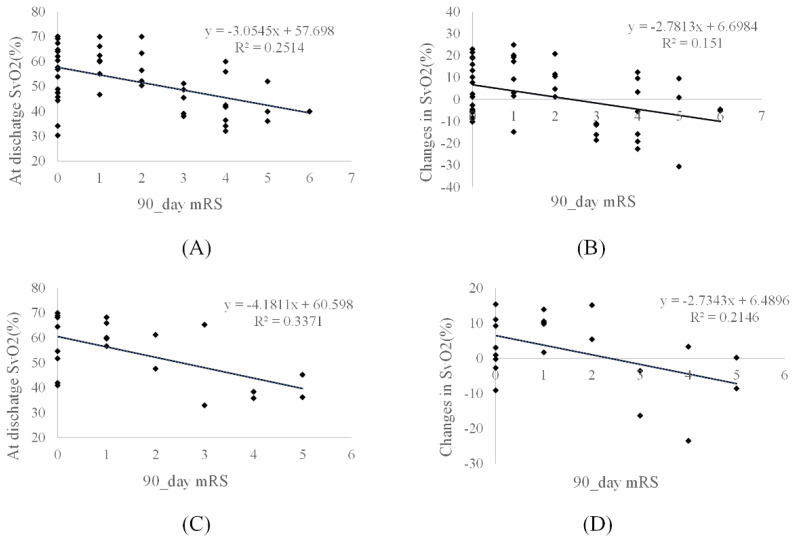
Scatter plots show the correlation between cerebral venous oxygen saturation (SvO2) and 90-day modified Rankin Scale (mRS) scores. The cerebral SvO2 (at follow-up and its changes) in time to maximum (Tmax) of more than (**A**,**B**) 4 s in 48 patients and (**C**,**D**) 6 s in 21 patients. Changes in SvO2: SvO2 at follow-up minus at baseline.

Pearson’s analysis demonstrated that the local cerebral SvO2 in hypoperfusion regions (Tmax > 4 s and 6 s) and its changes after treatment were negatively correlated with NIHSS scores (Table 4) and 90-day mRS scores (Figure 4). There was no correlation between cerebral SvO2 in the severe hypoperfusion and clinical scores based on Pearson’s and Spearman’s analyses (all *p* > 0.05).

### 3.4. Receiver Operating Characteristic Analysis

**Figure 5:**
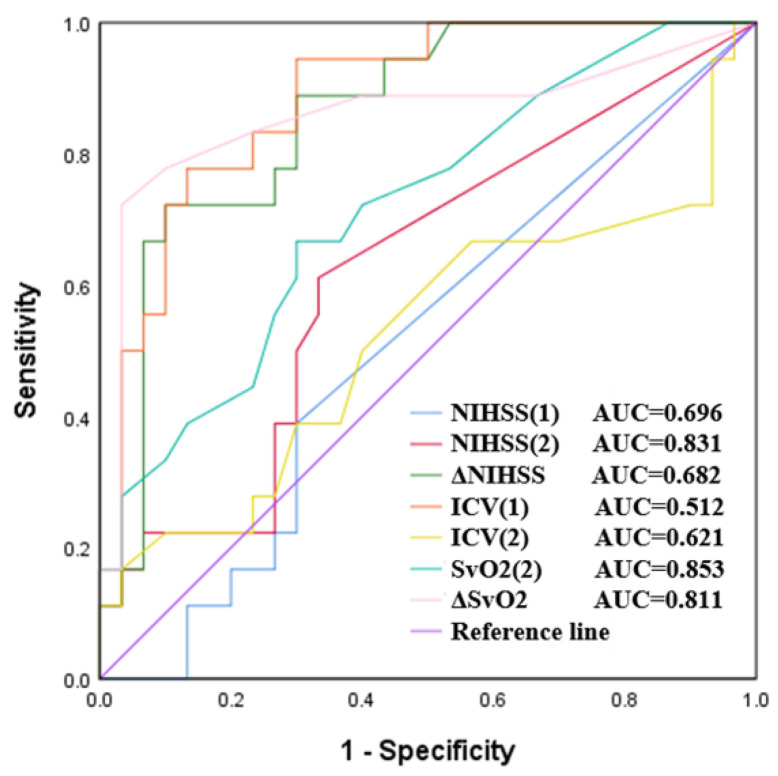
Receiver operating characteristic (ROC) curves of different indicators in predicting unfavorable clinical outcomes in all patients. ICV: ischemic core volume. (1): baseline. (2): follow-up. Δ: follow-up minus baseline.

There were significant differences between cerebral SvO2 in hypoperfusion (Tmax > 4 s) and NIHSS scores, ischemic core volume, and 90-day mRS (all *p* < 0.05). Cerebral SvO2 can be used as an independent indicator to predict the prognosis of patients. The ROC analysis demonstrated that cerebral SvO2 in hypoperfusion at follow-up had the highest AUC (0.853, 95% CI 0.743–0.962). It was higher than the follow-up ischemic core volume (AUC 0.621 95% CI 0.456–0.787), which was similar to the follow-up NIHSS scores (AUC 0.831, 95% CI 0.693–0.968) in predicting unfavorable clinical outcomes (Figure 5).

Table 5 summarizes the positive predictive value, negative predictive value, and diagnostic accuracy of different indicators in the diagnostic experiment. The ability to predict unfavorable clinical outcomes by cerebral SvO2 in hypoperfusion was higher than that by infarct volume. It was even higher than the NIHSS scores in terms of positive predictive value.

## 4. Discussion

In this paper, the cerebral SvO2 in different hypoperfusion regions and its changes after treatment were automatically and quantitatively measured by combining QSM images and baseline Tmax maps. Additionally, the decreased cerebral SvO2 of ischemic brain tissue was used to predict the unfavorable clinical outcomes of AIS patients. Moreover, this method allowed us to evaluate the ischemic tissue not only from the PWI-DWI mismatch but also from the oxygen metabolism mechanism. The cerebral SvO2 of hypoperfusion regions may have potential clinical values in evaluating the physiological state of brain tissue and a strategy of treatment selection in both acute and subacute stages.

The measurement of SvO2 in hypoperfusion regions of stroke patients was more suitable for routine clinical examination. In previous studies, researchers often measured cerebral SvO2 in the APCV regions based on QSM, and they found that the local cerebral SvO2 decreased by 16–44% after stroke [19]. However, the accuracy of APCV region mapping depends more on the experience of clinicians. In addition, to ensure no difference between different observers, 2–3 clinicians were usually required to evaluate the consistency after independent mapping in some studies [18,19,20]. Obviously, this could not meet the needs of clinical and big data analysis. Therefore, we proposed using the position information provided by Tmax images to calculate cerebral SvO2 in ischemic tissues and its changes after treatment. This also allowed us to evaluate the ischemic brain tissue from the oxygen metabolism mechanism in the clinic.

Due to the occlusion of the blood supply artery, when the cerebral oxygen supply could not meet the oxygen consumption, the deoxyhemoglobin content in the capillaries and drainage veins of ischemic tissue was relatively increased, resulting in a local oxygen extraction fraction [29]. QSM images showed that the number of drainage veins increased, and the vessels became thicker in the area of decreased perfusion, showing a very significant asymmetric high signal (APCV) compared with the non-infarcted side. This allowed us to measure the cerebral SvO2 based on QSM in stroke patients in a non-invasive way [30]. However, it does not mean that there was no change in oxygen saturation in the infarction hemisphere without APCV. It is probable that they could not be detected with QSM in a 1.5T magnet. We will further study the change of oxygen saturation in stroke patients under higher field magnets in the future.

In previous studies, NIHSS scores and ischemic core volume were commonly used to predict the prognosis of patients [31,32]. The high follow-up NIHSS scores and large infarct volume (baseline or final infarct volume increase) suggested the clinical outcome of AIS patients to be unfavorable. In the present study, the ROC curve analysis showed that the prediction accuracy of follow-up cerebral SvO2 was significantly higher than that of infarct volume, consistent with follow-up NIHSS scores. More interestingly, compared with NIHSS scores, without the participation of clinicians, cerebral SvO2 in the hypoperfusion calculated by performing automatic data processing had objective and repeatable advantages.

After intraarterial rt-PA treatment, the mean cerebral SvO2 of hypoperfusion tissue in the favorable clinical outcomes group was significantly improved (Tmax > 4 s, 6.37%, and Tmax > 6 s, 6.30%). On the contrary, the cerebral SvO2 decreased significantly in patients with unfavorable clinical outcomes (Tmax > 4 s, 6.68%, and Tmax > 6 s, 8.07%). These indicated that the changes in cerebral SvO2 in hypoperfusion regions could not only be used as an independent imaging parameter to predict clinical outcomes but also evaluate the intraarterial rt-PA treatment effect.

Moreover, previous studies also found that the clinical outcomes of some patients with a small infarct volume were unfavorable [33]. This may be caused by the choice of treatment scheme only being based on PWI-DWI mismatch ratio information [34]. Inappropriate treatment may result in unfavorable clinical outcomes for AIS patients. The cerebral SvO2 in ischemic tissue can provide more reference information for clinicians to make an accurate diagnosis and select appropriate treatment schemes.

There are several limitations to our study. First, this study was a single-center design with a small sample size, so there is some deviation in data statistics. Second, the collateralization could affect the measurement results of cerebral SvO2, which was not evaluated. Third, the value of baseline SvO2 in predicting unfavorable clinical outcomes was unclear. Fourth, patients with severe hypoperfusion or those who died within 90 days after discharge were not selected in this study. Further, the assessment of the role of cerebral SvO2 in hypoperfusion regions in different ischemic stroke subtypes (especially in lacunar stroke) represents indispensable research [35]. Moreover, whether cerebral SvO2 in hypoperfusion regions measured by QSM has advantages over other imaging approaches (such as CT perfusion, Arterial Spin Labeling perfusion) in predicting unfavorable clinical outcomes and evaluating treatment should be investigated.

## 5. Conclusions

This study demonstrated that the cerebral SvO2 changes in hypoperfusion after treatment measured by QSM and PWI sequences could reflect the activity of ischemic tissue. Cerebral SvO2 in hypoperfusion regions and its changes can be used as an independent imaging indicator to predict unfavorable clinical outcomes in AIS patients.

## Figures and Tables

**Table 1:** Parameters of the magnetic resonance imaging protocol.

Sequence	Matrix Size	Slices	TR ^4^ (ms)	TE ^5^ (ms)	Bandwidth (Hz/pixel)	FOV ^6^ (mm^2^)	Pixel Spacing (mm)	Thickness (mm)	Others
SWI ^1^	260 × 320	72	79	40	80	230	0.718, 0.718	1.6	
PWI ^2^	128 × 128	19	1520	32	1346	230	0.898, 0.898	5	measurements = 50
DWI ^3^	192 × 192	18	3600	102	964	230	1.198, 1.198	5	B = 1000 s/mm^2^

^1^ SWI: susceptibility-weighted imaging. ^2^ PWI: perfusion-weighted imaging. ^3^ DWI: diffusion-weighted imaging. ^4^ TR: repetition time. ^5^ TE: echo time. ^6^ FOV: field of view.

**Table 2:** Patient characteristics differences between favorable and unfavorable clinical outcome groups (*n* = 48).

Characteristics	Favorable (*n* = 30)	Unfavorable (*n* = 18)	*p*
Baseline			
Age(years)	69.6 ± 11.3	72.9 ± 7.6	0.232
Sex, male (%)	23 (76.7)	11 (61.1)	0.251
Risk factor			
Hypertension	24 (80.0)	13 (72.2)	0.535
Diabetes	6 (20.0)	8 (44.4)	0.071
Atrial fibrillation	10 (33.3)	4 (22.2)	0.412
NIHSS ^1^	7.0 ± 6.1	8.9 ± 6.1	0.322
MRP measurements ^2^			
Infarct side, right (%)	21 (70.0)	16 (88.9)	0.132
ICV (mL) ^3^	15.2 ± 31.1	8.3 ± 16.0	0.386
HPV (mL) ^4^	69.5 ± 103.94	87.5 ± 105.3	0.565
Follow-up			
Interval time (days)	11.3 ± 4.7	12.8 ± 4.8	0.299
NIHSS	1.6 ± 2.0	7.4 ± 5.2	0.000 *
ΔNIHSS ^5^	−5.4 ± 6.6	−1.5 ± 5.9	0.047 *
MRP measurements			
ICV (mL)	16.0 ± 35.4	38.3 ± 70.2	0.149
HPV (mL)	18.8 ± 42.0	69.9 ± 98.0	0.048 *

^1^ NIHSS: National Institutes of Health Stroke Scale. ^2^ MRP: magnetic resonance perfusion. ^3^ ICV: ischemic core volume. ^4^ HPV: hypoperfusion volume. ^5^ Δ: value measured at follow-up minus baseline. *: *p*-value < 0.05.

**Table 3:** The cerebral venous oxygen saturation in different regions of patients with favorable and unfavorable outcomes.

SvO2 ^1^(%)	Tmax > 4 s ^2^		Tmax > 6 s		Tmax > 8 s		Tmax > 10 s	
	(+) *n* = 30 ^3^	(−) *n* = 18 ^4^	(+) *n* = 15	(−) *n* = 6	(+) *n* = 13	(−) *n* = 4	(+) *n* = 13	(−) *n* = 4
Baseline	50.21 ± 9.28	50.77 ± 8.14	52.41 ± 9.50	50.39 ± 11.98	51.00 ± 9.08	47.79 ± 8.31	53.78 ± 12.89	47.56 ± 4.31
Follow-up	56.59 ± 10.24	42.39 ± 8.27	58.71 ± 9.63	42.31 ± 12.00	53.86 ± 11.43	45.96 ± 15.37	56.63 ± 10.89	49.59 ± 13.45
Changes	6.37 ± 11.37	−6.68 ± 13.04	6.30 ± 7.17	−8.07 ± 10.25	−2.86 ± 7.82	1.82 ± 13.06	−2.85 ± 8.33	−2.04 ± 12.94

^1^ SvO2: venous oxygen saturation. ^2^ Tmax: time to maximum peak. ^3^ (+): favorable clinical outcome. ^4^ (−): unfavorable clinical outcome.

**Table 4:** Relationship between cerebral venous oxygen saturation in different hypoperfusion regions and National Institutes of Health Stroke Scale scores.

SvO2	Tmax > 4 s (*n* = 48)	Tmax > 6 s (*n* = 21)	Tmax > 8 s (*n* = 17)	Tmax > 10 s (*n* = 17)
Baseline	−0.343 **	−0.483 *	−0.061	−0.329
Follow-up	−0.455 **	−0.610 **	−0.152	−0.110
Changes	−0.349 *	−0.552 **	−0.445	−0.358

*: *p*-value < 0.05. **: *p*-value < 0.01.

**Table 5:** Evaluation results of the clinical outcome prediction experiment for stroke patients.

Parameters	PPV (%) ^1^	NPV (%) ^2^	Accuracy (%)
NIHSS (1) ^3^	63.6	38.9	54.2
NIHSS (2)	82.9	92.3	85.4
ΔNIHSS	73.5	64.2	70.8
ICV (1)	64.7	42.9	58.3
ICV (2)	74.1	52.4	64.6
SvO2 (2)	91.7	66.7	79.2
ΔSvO2	84.6	63.6	75.0

^1^ PPV: positive predictive value. ^2^ NPV: negative predictive value. ^3^ (1): baseline. (2): follow-up.

## Data Availability

The data presented in this study are available on request from the corresponding author. The data are not publicly available due to ethical restrictions.
